# High CO_2_ enhances the competitive strength of seaweeds over corals

**DOI:** 10.1111/j.1461-0248.2010.01565.x

**Published:** 2011-02

**Authors:** Guillermo Diaz-Pulido, Marine Gouezo, Bronte Tilbrook, Sophie Dove, Kenneth R N Anthony

**Affiliations:** 1 Griffith School of Environment, Australian Rivers Institute and ARC Centre of Excellence for Coral Reef Studies, Nathan Campus, Griffith University170 Kessels Road, Brisbane, Nathan, Qld 4111, Australia; 2 Global Change Institute and ARC Centre of Excellence for Coral Reef Studies, The University of QueenslandSt Lucia, Brisbane, Qld 4072, Australia; 3 CSIRO Wealth from Oceans National Research FlagshipPO Box 1538, Hobart, Tas. 7001, Australia

**Keywords:** Carbon dioxide, coral reefs, coral–algal competition, macroalgae, ocean acidification

## Abstract

Space competition between corals and seaweeds is an important ecological process underlying coral-reef dynamics. Processes promoting seaweed growth and survival, such as herbivore overfishing and eutrophication, can lead to local reef degradation. Here, we present the case that increasing concentrations of atmospheric CO_2_ may be an additional process driving a shift from corals to seaweeds on reefs. Coral (*Acropora intermedia*) mortality in contact with a common coral-reef seaweed (*Lobophora papenfussii*) increased two- to threefold between background CO_2_ (400 ppm) and highest level projected for late 21st century (1140 ppm). The strong interaction between CO_2_ and seaweeds on coral mortality was most likely attributable to a chemical competitive mechanism, as control corals with algal mimics showed no mortality. Our results suggest that coral (*Acropora*) reefs may become increasingly susceptible to seaweed proliferation under ocean acidification, and processes regulating algal abundance (e.g. herbivory) will play an increasingly important role in maintaining coral abundance.

## Introduction

Corals and macroalgae are among the dominant benthic groups on coral reefs, and their relative abundance and competition are often used as indicators of reef status (Mumby *et al.* 2007). Environmental and anthropogenic processes that drive the competitive interactions, particularly space competition, between corals and seaweeds (fleshy macroalgae) have profound consequences for the dynamics and resilience of reef communities. Well-known processes that can shift reef communities from coral- to seaweed-dominated regimes include the loss of herbivore grazing through overfishing or disease (Hughes *et al.* 2007), eutrophication via enhanced algal growth (Lapointe 1999) and coral mortality by releasing macroalgae from space competition with corals (Diaz-Pulido *et al.* 2009). Increasing concentrations of atmospheric carbon dioxide, CO_2_, and associated ocean acidification is a growing threat to coral reefs globally (Caldeira & Wickett 2003; Hoegh-Guldberg *et al.* 2007), as it can lead to reduced growth rates (e.g. calcification) of corals and other marine calcifiers (Kleypas *et al.* 2006; Anthony *et al.* 2008). Seaweeds show varying responses to ocean acidification, including positive, neutral or negative effects (Israel & Hophy 2002; Diaz-Pulido *et al.* 2007; Hall-Spencer *et al.* 2008; Wu *et al.* 2008), but the role of CO_2_ enrichment in driving coral–algal interactions and the consequence for reef resilience is unknown (Harley *et al.* 2006; Hoegh-Guldberg *et al.* 2007).

Here, we test the hypothesis that ocean acidification shifts the competitive interaction between corals and seaweeds in favour of the seaweeds. We build this hypothesis on the following two premises: First, high *p*CO_2_ leads to declining aragonite saturation state (Caldeira & Wickett 2003), which, as a functional consequence, leads to reduced rates of coral growth (Langdon & Atkinson 2005; Kleypas *et al.* 2006; Silverman *et al.* 2009), potentially lowering their ability for space competition. Second, growth of subtidal seaweeds is likely to increase as a function of *p*CO_2_ (Kübler *et al.* 1999; Diaz-Pulido *et al.* 2007; Hall-Spencer *et al.* 2008), enhancing the seaweed’s ability to overgrow and kill neighbouring corals (e.g. see Jompa & McCook 2002). To analyze this hypothesis experimentally, we compared the survival profiles of the branching coral *Acropora intermedia* (Brook) grown in isolation and in competition with the brown fleshy seaweed *Lobophora papenfussii* (Taylor) Farghaly in a flow-through aquarium system with controlled CO_2_-dosing regimes for an 8-week period. The coral genus *Acropora* plays fundamental ecological, geological and physical roles in the Great Barrier Reef (GBR) as it is the most abundant hard coral group and a major reef builder in the region (Hutchings *et al.* 2008). Species of the macroalgal genus *Lobophora* are ecologically important in both shallow and deep reefs due to their high abundance, and have also been implicated in the degradation of Caribbean (Mumby *et al.* 2007) and GBR (Diaz-Pulido *et al.* 2009) reefs. Under normal undisturbed conditions, the seaweed commonly grows at the dead base of coral branches, but away (*c*. > 5–10 cm) from the healthy coral tissue. The algae’s ability to grow beyond the base of healthy coral branches is limited, due to competitive inhibition by the corals or the removal of algal tissue by herbivores. However, the interaction between branching corals and the seaweed *Lobophora* is competitive, with both corals and seaweeds inhibiting each other (Jompa & McCook 2002; Diaz-Pulido *et al.* 2009). In this study, we show that increased *p*CO_2_/reduced pH, representing the ocean-acidification range projected for this century, enhances the ability of seaweeds to kill corals, potentially shifting the competitive interaction between corals and seaweeds in favour of seaweeds.

## Materials and methods

### Experimental design and CO_2_ system

We tested the combined effects of ocean acidification and algal–coral competition on coral survivorship and growth through simultaneous manipulation of competitors and CO_2_ levels in an orthogonal, multifactorial experiment carried out on Heron Island Research Station (HIRS), southern GBR. The experiment was conducted during May and June, the Austral autumn/winter, of 2009. We used branches of the coral *A. intermedia* and thalli of the seaweed *L. papenfussii*, which are both abundant in reef fronts and upper slopes on GBR reefs. Seaweeds and corals were collected from the reef slope at Coral Gardens (23°26.698′ S, 151°54.533′ E), Heron Reef. The competitor treatment consisted of three manipulation groups: (i) isolated live coral branches, 10-cm long; (ii) pairs of competing corals and seaweeds, in which seaweeds (thalli of *c*. 5 cm × 5 cm planar area) were attached to the base of the coral branch; and (iii) isolated seaweeds, attached to dead coral. An additional isolated control group involved live coral branches with algal mimics (latex thalli) attached to the coral base, and was used to partly discern whether interaction outcomes were due to mechanical or biological/chemical effects on the coral. Due to space and CO_2_-treated seawater restrictions, the algal mimic treatment was conducted only for the 400-ppm control treatment. Both live and dead coral branches were suspended from their apical corallites using thin monofilament line to minimize coral and algal contact with other surfaces. Coral and algal specimens were acclimatized in tanks with running seawater during 10 days prior to the experiment.

The CO_2_ experiment involved four CO_2_-dosing regimes to cover the range of historical and potentially future ocean-acidification conditions. We used CO_2_ target values of 300 (pH target: 8.10–8.20), 400 (pH: 8.00–8.10), 560 (pH: 7.85–7.95) and 1140 ppm (pH: 7.60–7.70), representing pre-industrial, present-day, and projected mid-century and late-century CO_2_ levels under the A1FI scenario by the IPCC (Meehl *et al.* 2007; Meinshausen *et al.* 2009; Solomon *et al.* 2009). The CO_2_ control system (Aquatronica-AEB Technologies, Cavriago, Italy) used computer-operated solenoid valves to regulate the amount of CO_2_ injected into the seawater, based on pH levels measured continuously in 200-L mixing tanks (one mixing tank per CO_2_ treatment) using Mettler-Toledo polarographic sensors (see Anthony *et al.* 2008, for details). Sumps were connected to the outdoor flow-through aquarium system at HIRS, which uses unfiltered seawater pumped directly from the reef flat. To dampen diurnal pH fluctuations (7.9–8.4) of the pH from the reef flat (caused by benthic carbon fluxes), and better simulate the more stable pH regime on the outer reef slope where both corals and macroalgae were collected, low and high pH extremes of the control were prevented by dosing CO_2_ when pH exceeded 8.2 and scrubbing CO_2_ (see below) when pH fell below 8.0. CO_2_ scrubbing was used to generate a pre-industrial CO_2_ regime (*c*. 300 ppm), using the procedure described in Reynaud *et al.* (2003). Briefly, air was filtered through a column (2 m tall × 5 cm diameter) of soda lime before being injected into the water in the mixing tank for the pre-industrial treatment (and for the control tank when pH dropped below 8). Individual experimental tanks (10-L plastic aquaria) were randomly assigned to treatments and were continuously fed with water from the treated sump with a flow of 2–3 L min^−1^. Each tank had a small powerhead for water circulation. Each CO_2_ level had two tanks per treatment combination, each with 10 subsamples and a total of 160 seaweeds and 180 corals. To mimic the light field at the native habitat (6 ± 3 m depth) of both corals and algae, shade screens were used to reduce the natural sunlight to average noon levels of 320 (min: 100, max: 1063) μmol m^−2^ s^−1^. Tanks were cleaned every 2–3 days of filamentous algae and inspected for the presence of mesograzers.

### Water chemistry analyses

Samples for water chemistry analyses were collected during the course of the experiment (see details in Table 1). Total dissolved inorganic carbon (TCO_2_) was measured coulometrically and total alkalinity (TA) analyses were made by open-cell potentiometric titration (Dickson *et al.* 2007), using seawater samples collected from the sumps every 6 h during a 24-h period. The *p*CO_2_, aragonite saturation state (Ω_Arag_) and pH on the total seawater scale were calculated for 24–25 °C from the TCO_2_ and TA values (Lewis & Wallace 1998). Dissolved organic carbon (DOC) was measured by high-temperature combustion (680 °C) using a Shimadzu TOC-5000A carbon analyzer (Shimadzu Corporation, Kyoto, Japan), and dissolved inorganic nutrients were determined by standard wet chemical methods using a segmented flow analyzer. DOC and nutrients data were determined using seawater from the experimental tanks collected during the day.

**Table 1:** Summary of values for water chemistry parameters for CO_2_ treatment levels

Treatments	pH	pCO_2_ (ppm)	TA (μmol kg^−1^)	TCO_2_ (μmol kg^−1^)	Ω_Arag_	DOC (mg L^−1^)	NH_4_ (μmol L^−1^)	NO_2_ (μmol L^−1^)	NO_3_ (μmol L^−1^)	PO_4_ (μmol L^−1^)
300	8.12 (0.02)	305 (11)	2193 (13)	1882 (16)	3.37 (0.06)	0.65 (0.02)	0.037 (0.023)	0.038 (0.005)	0.450 (0.020)	0.237 (0.007)
400	8.02 (0.02)	402 (20)	2170 (27)	1918 (29)	2.78 (0.07)	0.68 (0.01)	0.020 (0.020)	0.032 (0.009)	0.460 (0.058)	0.238 (0.010)
560	7.85 (0.01)	564 (12)	2208 (22)	2012 (17)	2.26 (0.07)	0.69 (0.01)	0.072 (0.045)	0.036 (0.010)	0.524 (0.019)	0.254 (0.007)
1140	7.63 (0.02)	1140 (52)	2212 (20)	2123 (24)	1.32 (0.04)	0.71 (0.03)	0.070 (0.045)	0.033 (0.002)	0.545 (0.024)	0.252 (0.007)

pH, pCO_2_ (partial pressure CO_2_), TA (total alkalinity), TCO_2_ (total dissolved inorganic carbon) and Ω_Arag_ (aragonite saturation state) values are means of eight replicates (SEM). DOC (dissolved organic carbon), NH_4_, NO_2_, NO_3_ and PO_4_ are means of six replicates (SEM).

### Response variables and data analyses

A census of coral survivorship was conducted daily throughout the course of the experiment. Coral branches were considered dead when at least half of the branch was devoid of tissue. Dead corals were subsequently removed from the experimental tanks. Growth of both corals and macroalgae was measured to quantify the level of competition. At the beginning of the experiment, a marker (plastic cable tie, 1 mm thick) was tied to the bottom part of each coral branch or rubble as a reference point for growth measurements. Coral growth (as linear extension) was measured as the increase in the distance from the coral tip to the cable tie. Algal growth (as marginal growth) was measured as the extension of the blade margin relative to the reference point (Jompa & McCook 2002). Photographs of each specimen were taken at the beginning and end of the experiment, and growth estimates were made using ImageJ software (http://rsbweb.nih.gov/ij/).

Coral survival was analyzed using an exponential failure-time (hazard) model with CO_2_ and tanks as factors (Muenchow 1986). Effects of CO_2_ and competition on coral and seaweed growth were analyzed using a factorial, nested anova, with competitor and CO_2_ levels as fixed factors, and tanks as replicates. Specimens were nested within tanks. However, because the tank factor was non-significant (*P*> 0.25, data not shown), tanks were pooled in subsequent analyses and specimens were used as replicates (Underwood 1997), hence increasing the power of the analysis. Data from the latter analyses are presented here, followed by *post hoc* Student–Newman–Keuls (SNK) tests. To minimize the risks of overlooking small CO_2_ effects (i.e. Type II errors), CO_2_ effects on seaweed growth were specifically tested within the isolated seaweed treatment (in the absence of the corals) using a one-way anova. Some algal and coral specimens were lost during the course of the 8-week experiment, reducing the degrees of freedom in the analyses. Data were tested for homogeneity of variance (Cochran’s test), outliers and normality of residuals.

## Results

**Figure 1:**
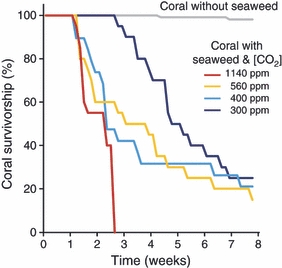
Coral mortality as a function of time, CO_2_ level and seaweed competition. The initial population size for each treatment group was 20 corals. The curve of coral without seaweed is the average of the survivorship rate of isolated corals (with no algal contact) across all CO_2_ treatments and in contact with plastic seaweeds.

**Figure 2:**
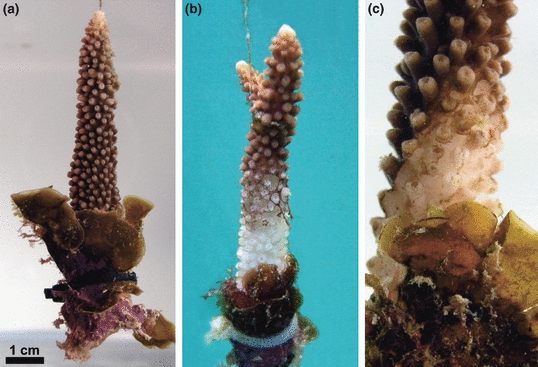
Coral mortality caused by seaweeds in experimental coral–algal pairs exposed to high CO_2_ levels. (a) Competing corals and seaweeds of the species *Acropora intermedia* and *Lobophora papenfussii* at the beginning of the experiment. (b) and (c) Coral branches with advanced tissue mortality caused by the seaweed. Coral tissue disintegration occurred rapidly in the high CO_2_ treatment. Coral white areas show bare coral skeleton devoid of coral tissue.

Elevated *p*CO_2_ strongly increased mortality rates of corals in contact with seaweeds. In the highest *p*CO_2_ treatment (1140 ppm), very high mortality rates (5–10% per day) set in after 1 week and the entire coral population in this treatment died within 3 weeks (Fig. 1). In contrast, for the low *p*CO_2_ treatment (300 ppm, pre-industrial), high coral mortality set in after 3 weeks and continued at a slower rate than at the high CO_2_ level. Corals in the 400 and 560 ppm treatments showed high initial mortality rates (after 2–3 weeks), but ended with 20–30% survival after 8 weeks, similar to that of the pre-industrial group. The proportional hazard model indicated that the CO_2_ level was significantly correlated with coral mortality (*P*< 0.001), although the absolute coral mortality rate (in the treatment in contact with seaweeds) of all but the highest level of CO_2_ was similar at week 8. Isolated coral branches (control corals with no seaweeds) showed < 5% mortality, regardless of the CO_2_ level. Mortality of corals with algal mimics (thin latex thalli) attached was also negligible (< 5%, Fig. 1). Coral mortality caused by the presence of seaweeds occurred at the interface between the algal thalli and the coral (Fig. 2) and was not preceded by tissue bleaching. Following the onset of coral tissue necrosis in the contact zone, tissue disintegration progressed up the branch at a rate of 2–3 cm day^−1^.

**Figure 3:**
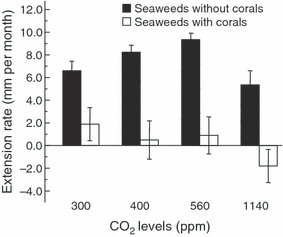
Growth (marginal extension) response of seaweed *Lobophora papenfussii* to increased CO_2_ concentrations and presence of coral competitor (*Acropora intermedia*). Data are means ± SEM; *n* = 15–20.

**Figure 4:**
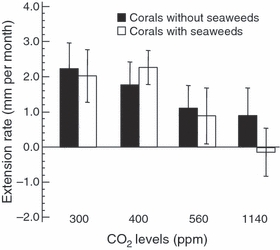
Growth (linear extension) response of coral *Acropora intermedia* to increased CO_2_ concentrations and presence of seaweed competitor (*Lobophora papenfussii*). Data are means ± SEM; *n* = 14–20.

The rate of marginal extension of seaweed thalli was significantly affected by both competitor and *p*CO_2_ treatments. Seaweed growth rate was higher in the absence of live corals (i.e. algal controls growing on dead coral rubble) compared with the treatment where the alga was in contact with live coral (*P*< 0.001; Table 2; Fig. 3). Localized damage to the margin of the algal blades was observed at the interface with the coral. The effect of CO_2_ on algal growth was significant (*P*= 0.046; Table 2), but smaller than the effect of the competitor (Fig. 3). SNK *post hoc* tests in the overall analyses indicated that the algal growth rate was lower in the highest *p*CO_2_ treatment than at any other CO_2_ level (Table 2), whereas there was no significant difference between growth rates at 300, 400 and 560 ppm *p*CO_2_. Because the presence of the competing coral reduced algal growth rates (potentially masking small CO_2_ effects), *p*CO_2_ effects on seaweed growth were specifically tested within the isolated seaweed treatment. Results of this analysis were significant (*P*= 0.006) and SNK *post hoc* test (Table 2 footnote) showed that growth rates of isolated seaweed thalli at the 400- and 560-ppm CO_2_ level were significantly higher (24% and 40% respectively) than that in the low pre-industrial CO_2_ treatment. Algal growth rate, however, declined to pre-industrial levels at the highest *p*CO_2_ (Table 2 footnote; Fig. 3). The growth rate of seaweeds in the presence of corals was negligible and did not vary significantly across *p*CO_2_ treatments (*P*= 0.439; Fig. 3). In contrast, the growth rate of corals (expressed as linear extension) in isolation and in the presence of seaweeds declined dramatically and monotonically with increasing *p*CO_2_ (*P*= 0.039), reaching negative values at the highest CO_2_ concentration (Table 2; Fig. 4). There was no significant difference (*P*= 0.622) in the growth rates of isolated corals vs. corals in competition.

**Table 2:** Factorial anovas for the effects of CO_2_ levels and coral–algal competition on the growth of seaweeds and corals

Source of variation	d.f.	MS	*F*	*P*	Conclusion – SNK test
Seaweeds					
CO_2_	3	70.08	2.74	0.046	(300 = 400 = 560) > 1140*
Competition	1	1710.71	66.75	< 0.001	Isolated alga > alga with coral
CO_2_ × Competition	3	22.86	0.89	0.447	n.s.
Error	133	25.63			
Corals					
CO_2_	3	23.021	2.863	0.039	300 = 400 > 560 = 1140
Competition	1	1.963	0.244	0.622	n.s.
CO_2_ × Competition	3	3.175	0.395	0.757	n.s.
Error	127	8.041			

MS, mean square; SNK, Student–Newman–Keuls test; n.s., not significant.

* We also specifically tested for CO_2_ effects on seaweed growth within the isolated seaweed treatment. The results of this test indicated: anova, *P*= 0.006; SNK: (400 = 560) > 1140, 560 > 300, 400 > (300 = 1140). anova for the effects of CO_2_ on seaweed growth within the seaweed-coral treatment was not significant (*P*= 0.439).

## Discussion

It is now well documented that rising atmospheric CO_2_ levels and the consequent changes in ocean carbon chemistry (ocean acidification) have profound implications for the physiology of many marine organisms (Raven 2005; Kleypas *et al.* 2006; Anthony *et al.* 2008; Pelejero *et al.* 2010). However, the impacts of ocean acidification on marine ecological interactions are largely unknown (Harley *et al.* 2006; Doney *et al.* 2009). The results of this study provide novel empirical evidence that ocean acidification alters the competitive interactions between two species of reef organisms belonging to the most abundant benthic groups on coral reefs: hard corals and benthic algae. Corals and algae compete for space and light, and fleshy macroalgae (seaweeds) may out-compete corals (McCook *et al.* 2001; Nugues *et al.* 2004b; Smith *et al.* 2006), particularly under situations of low herbivory (Jompa & McCook 2002; Rasher & Hay 2010). In our experiments, we demonstrate that ocean acidification enhances the ability of a common seaweed (*Lobophora*) to kill and potentially out-compete a coral (*Acropora*). Only corals in contact with live seaweeds showed significant mortality, and mortality was exacerbated by elevated *p*CO_2_. This indicates that coral mortality can be attributed to the presence of, and interaction with, seaweeds. Accordingly, increased coral mortality with increasing *p*CO_2_ is therefore likely to be a consequence of CO_2_, enhancing the competitive strength of the seaweeds.

The mechanism by which increased *p*CO_2_ enhanced coral mortality at the interface between the seaweed and the coral tissues could not be deduced from our observations or data. However, our experiment may suggest that mortality was caused by a chemical or biological rather than a physical (e.g. shading or abrasion) effect, as coral mortality occurred only when seaweeds, and not inert algal mimic (latex blades), were present. Although the algal mimic treatment was only conducted for the control CO_2_ level (potentially limiting the interpretation of interactions between algal presence and CO_2_ level), coral mortality across all CO_2_ treatments took place before the seaweed could overgrow the corals (as seaweeds showed limited marginal growth when in contact with the corals, possibly caused by inhibition by the coral polyps when still alive). This strongly suggests that coral mortality was caused by a chemical/biological interaction process. We propose two likely mechanisms: First, increased allelochemicals released by the seaweed can kill the coral directly (e.g. McCook *et al.* 2001; Rasher & Hay 2010). *Lobophora* has been shown to produce a range of secondary metabolites that are effective as chemical defenses (Rasher & Hay 2010), particularly when plants are stressed (Arnold & Targett 2000), for example by contact with corals (e.g. Nugues *et al.* 2004a). Further, there is evidence in terrestrial C_3_-plants that increased CO_2_ concentrations can lead to increased production of carbon-based allelochemicals due to augmented production of carbohydrates (Bidart-Bouzat & Imeh-Nathaniel 2008). Second, increasing leakage of DOC from the seaweeds can cause an increase in microbial growth on the surface of the corals, as suggested by Smith *et al.* (2006). However, the DOC level was lower in the algal and coral–algal competition treatment than in that of the isolated corals (anova, *P*= 0.046), providing no support for this hypothesis.

The increase in competitive strength of seaweeds over corals under elevated CO_2_ was also supported by a comparison of growth patterns for seaweeds and corals. Specifically, the growth of seaweed blades in the absence of corals increased with rising *p*CO_2_ (although algal growth declined to pre-industrial levels at the highest *p*CO_2_). These results are consistent with studies, particularly on red seaweeds, demonstrating enhanced algal growth rates with moderate enhancement of *p*CO_2_ (Gao *et al.* 1991, 1993; Kübler *et al.* 1999). However, there is also other evidence indicating that growth of benthic algae does not respond to increased *p*CO_2_ because algae are able to use the carbon from the abundant bicarbonate (HCO_3_^−^) pool in seawater by a series of cellular CO_2_-concentrating mechanisms (CCMs; Israel & Hophy 2002; Raven *et al.* 2005). Although it has been suggested that the Caribbean seaweed *Lobophora variegata* possesses CCMs (Enríquez & Rodríguez-Román 2006), growth of the experimental seaweed *L. papenfussii* in this study was enhanced by moderate CO_2_ enrichment (< 560 ppm). On the other hand, growth rates of corals declined with increased *p*CO_2_ (lowered pH), regardless of the competition treatment. Reduction of coral growth may be related to the lowering of the aragonite saturation state (Table 1) associated with a reduction of the amount of carbonate ions available for coral calcification as pH levels decrease. Stress from acidosis and/or damage to the photoprotective mechanisms in corals have also been suggested to occur under ocean-acidification conditions (Anthony *et al.* 2008; Crawley *et al.* 2010). The increase in seaweed growth rate in conjunction with a decline in coral growth and enhanced coral mortality as *p*CO_2_ increased in this study strongly suggest that corals could become increasingly disadvantaged in space competition with macroalgae as ocean acidification intensifies.

On the other hand, the presence of the coral competitor inhibited the rate of marginal growth of seaweeds. This suggests that the seaweed may have reduced ability to overgrow live coral tissue and that the corals have some potential to reduce algal overgrowth (see also de Ruyter van Steveninck *et al.* 1988; Nugues *et al.* 2004a). However, because algae caused significant mortality of coral tissue, particularly at the high *p*CO_2_ treatment, any potential inhibition of seaweed growth by coral may be overwhelmed by the negative effect of seaweeds on coral survivorship. It is likely that the seaweeds would have been released from space competition with the coral following coral tissue mortality and grown unrestrictedly if dead coral–algal pairs had not been removed from the tanks immediately after the corals died (to prevent potential water quality deterioration). Therefore, increased algal growth following coral mortality could not be demonstrated in our study. However, previous studies have shown increased algal growth and colonization following coral disturbances (Jompa & McCook 2002; Diaz-Pulido *et al.* 2009).

The enhanced ability of *L. papenfussii* to kill *A. intermedia* and the increase in competitive strength of the seaweed over the corals as a function of CO_2_ enrichment suggest that future reefs may become more likely to undergo coral–algal phase shifts than predicted previously (Hoegh-Guldberg *et al.* 2007; Mumby *et al.* 2007; Maynard *et al.* 2010). We acknowledge that by using only one species pair, we are not accounting for the high diversity of coral and algal species in tropical reefs, or the variety in mechanisms by which they compete (McCook *et al.* 2001; Nugues *et al.* 2004b; Smith *et al.* 2006; Rasher & Hay 2010). However, as these two genera co-occur on the same reef habitat (although the *L. papenfussii* generally grow centimetres away from healthy coral tissue) and are widespread on coral reefs globally, our results may have wide implications for a large number of reef habitats. Our findings also suggest that reef community dynamics will be altered by increased mortality risk of adult corals. In particular, high abundance of various types of seaweeds can impede coral population recovery following disturbances and reduce the resilience of the reef ecosystem by hindering coral settlement and recruitment (Diaz-Pulido *et al.* 2010) and the survival of juvenile corals (Box & Mumby 2007; Birrell *et al.* 2008). For example, under conditions of reduced herbivory, the alga *Lobophora* significantly decreased the growth of juvenile corals in the Caribbean (Box & Mumby 2007), whereas in the GBR, the presence of *Lobophora* around the branches of the coral colonies caused significant coral tissue mortality (Jompa & McCook 2002). Therefore, processes that regulate the abundance and type of seaweeds may be increasingly important under rising CO_2_. For instance, grazing by herbivores, maintenance of the abundance and species diversity of herbivorous functional groups, and controls on water quality will become more and more critical to reduce macroalgal dominance and maintaining coral reef resilience (Bellwood *et al.* 2004; Hughes *et al.* 2007; Mumby *et al.* 2007; Burkepile & Hay 2008; Rasher & Hay 2010). However, the extent to which high herbivore abundance and diversity can control *Lobophora* proliferations and phase shifts from *Acropora* to *Lobophora* on some coral reefs in the future is uncertain, as recent evidence shows that *Lobophora* spp. may be much less susceptible to herbivory than other reef seaweeds (Bennett *et al.* 2010; but see Jompa & McCook 2002). Further, some inshore reefs of the central GBR have experienced persistent coral –*Lobophora* phase shifts (> 7 years), in the absence of fishing pressure on herbivorous fishes and at moderate water quality (Cheal *et al.* 2010). This suggests that even ambient levels of grazing may be inadequate to reduce the strength of spatial competition of *Lobophora* over corals, particularly after disturbances, potentially perpetuating phase shifts under ocean-acidification scenarios. Physical disturbances such as those caused by storm activities and algal seasonality may also play important roles in controlling the abundance of seaweeds in the future (e.g. Diaz-Pulido *et al.* 2009). Our experiment provides a timely example of how ocean acidification affects not only the physiological processes of reef organisms, but also the ecological interactions between species.
